# Genetically Engineered CLDN18.2 CAR-T Cells Expressing Synthetic PD1/CD28 Fusion Receptors Produced Using a Lentiviral Vector

**DOI:** 10.1007/s12275-024-00133-0

**Published:** 2024-05-03

**Authors:** Heon Ju Lee, Seo Jin Hwang, Eun Hee Jeong, Mi Hee Chang

**Affiliations:** CARBio Therapeutics Co., Ltd., Cheongju, 28160 Republic of Korea

**Keywords:** Synthetic PD1/CD28 receptor, CAR-T, Claudin18.2, Lentiviral vector, Gastric cancer

## Abstract

This study aimed to develop synthetic Claudin18.2 (CLDN18.2) chimeric antigen receptor (CAR)-T (CAR-T) cells as a treatment for advanced gastric cancer using lentiviral vector genetic engineering technology that targets the CLDN18.2 antigen and simultaneously overcomes the immunosuppressive environment caused by programmed cell death protein 1 (PD-1). Synthetic CAR T cells are a promising approach in cancer immunotherapy but face many challenges in solid tumors. One of the major problems is immunosuppression caused by PD-1. CLDN18.2, a gastric-specific membrane protein, is considered a potential therapeutic target for gastric and other cancers. In our study, CLDN18.2 CAR was a second-generation CAR with inducible T-cell costimulatory (CD278), and CLDN18.2-PD1/CD28 CAR was a third-generation CAR, wherein the synthetic PD1/CD28 chimeric-switch receptor (CSR) was added to the second-generation CAR. In vitro, we detected the secretion levels of different cytokines and the killing ability of CAR-T cells. We found that the secretion of cytokines such as interferon-gamma (IFN-γ) and tumor necrosis factor-alpha (TNF-α) secreted by three types of CAR-T cells was increased, and the killing ability against CLDN18.2-positive GC cells was enhanced. In vivo, we established a xenograft GC model and observed the antitumor effects and off-target toxicity of CAR-T cells. These results support that synthetic anti-CLDN18.2 CAR-T cells have antitumor effect and anti-CLDN18.2-PD1/CD28 CAR could provide a promising design strategy to improve the efficacy of CAR-T cells in advanced gastric cancer.

## Introduction

The long-standing dream of curing incurable diseases is gradually being realized, starting with a cure for blood cancer using chimeric antigen receptor (CAR)-T (CAR-T) gene therapy (June & Sadelain, 2018). Gene therapy, a field of synthetic biology, is a cutting-edge technology that corrects or treats diseases by synthesizing therapeutic genes and introducing them into the human body using delivery systems. In the field of CAR-T gene therapy, the United States was the first to conduct clinical trials in 1990 and began to announce clinical results in earnest in 2006. The United States and China are developing the most new drugs, leading clinical trials worldwide, followed by the United Kingdom and Germany (Maus & June, 2016).

Vectors used in gene therapy approved by the US Food and Drug Administration include viral and nonviral vectors. Viral vectors include lentiviruses, adenoviruses, retroviruses, adeno-associated viruses, and vaccinia viruses. Nonviral vectors include chemical delivery methods, such as plasmids, gold nanoparticles, lipid nanoparticles, and N-acetylgalactosamine, and physical delivery methods, such as microinjection and electroporation (Gill & June, 2015; Zhou et al., 2016). Viral vectors are commonly used in gene therapy development. Among them, self-inactivating lentiviral vectors, the safest third-generation lentiviruses, have recently been used in many clinical trials. It introduces four types of plasmid DNAs into production cells: one type of transfer plasmid DNA, two types of packaging plasmid DNAs, and one type of envelope plasmid DNA (VSV-G) (Milone & O’Doherty, 2018). Recently, as a more convenient method, there has been a trend to commercially use integrated, stable producer cell lines containing the above four plasmid DNAs without the need to separately produce transient lentiviral vectors containing each of these plasmids.

Genetically synthetic CAR-T cell therapy, which is attracting attention as a next-generation immunotherapy, is an ex vivo gene therapy developed by transducing lentiviral vectors containing transgenes into mature T cells. CAR-T cells are engineered to be specific for an antigen that is expressed on tumor cells but not on healthy cells (Srivastava & Riddell, 2015). Novartis’ Kymriah, the first CAR-T cell treatment, was developed to efficiently destroy cancer cells by introducing a receptor gene into T cells that recognizes the CD-19 antigen expressed on the cancer cells of patients with B-cell malignancies (Gill & June, 2015; Maus & June, 2016). Since the US Food and Drug Administration approved Kymriah in 2017, numerous studies have been conducted on CAR-T treatments (Mohanty et al., 2019). To date, the following six CAR-T gene therapies have been approved only in the field of incurable blood cancer, including CD19 and B-cell maturation antigen (BCMA) targets (Mazinani & Rahbarizadeh, 2022): Kymriah™ (B-cell acute lymphoblastic leukemia [ALL] and B-cell non-Hodgkin lymphoma [NHL]), Yescarta™ (NHL and follicular lymphoma), Tecartus™ (Mantle cell lymphoma and ALL), Breyanzy™ (NHL), Abecma™ (multiple myeloma), and Carvykte™ (multiple myeloma).

Multifunctional CAR-T cell candidates are being developed as CAR-T treatments for incurable blood cancer, solid cancer, and the autoimmune disease systemic lupus erythematosus (Patel et al., 2020; Tahir, 2018). As a treatment for blood cancers, CAR-T cells using the following antigen receptors are being developed: CD19 (Fousek et al., 2021), CD20 (Shah et al., 2019), CD22 (Fry et al., 2018; Ruella et al., 2017), CD19/20 (Zah et al., 2016), CD19/22 (Qin et al., 2018), CD19/123 (Ruella et al., 2016), CD19/20/22 (Bielamowicz et al., 2018; Fousek et al., 2021; Schneider et al., 2021), CD43, CD47 (Jiang et al., 2021a, b, c; Yamada-Hunter et al., 2023), CD33 (Mardiana & Gill, 2020), CD123 (Thokala et al., 2016), NY-ESO-1 (Wang & Wang, 2017), BCMA (Friedman et al., 2018); CS1/BCMA (Chen et al., 2018), CD19/BCMA (Marofi et al., 2021), FLT3 (Wang et al., 2018), tyrosine-protein kinase transmembrane receptor (Daneshmanesh et al., 2008; Hashem Boroojerdi, et al., 2020; Jiang et al., 2021a, b, c), etc. As a treatment for solid cancers, CAR-T cells using the following antigen receptors are being developed: epidermal growth factor receptor (EGFR) (Miao et al., 2021), EGFR vIII (Johnson et al., 2015), human epidermal growth factor receptor 2 (Candas-Green et al., 2020), mesothelin (Morello et al., 2016; Wang et al., 2021), prostate stem cell antigen (Wu et al., 2020), prostate-specific membrane antigen (Alzubi et al., 2020), mucin (Muc)1 (Dréau et al., 2019; Mei et al., 2020), Muc16 (Li & Wang, 2020), CD44 (Wang et al., 2020), CD47 (Beckett et al., 2023; Golubovskaya et al., 2017; Shu et al., 2021), CD70 (Jin et al., 2018; Yang et al., 2020), CD126 (Mishra et al., 2021), CD147 (Sakamoto et al., 2021; Tseng et al., 2020; Zheng et al., 2022), tyrosine-protein kinase transmembrane receptor (Zhao et al., 2021), mesenchymal-epithelial transition factor (Chen et al., 2021; Jiang et al., 2021a, b, c), carcinoembryonic antigen, glypican 3 (Li et al., 2023; Wu et al., 2019), disialoganglioside (Bates et al., 2021), Claudin18.2 (CLDN18.2), intercellular adhesion molecule 1 (Jung et al., 2020), etc. Moreover, to enhance the efficacy of CAR-T treatment and improve the tumor microenvironment, which are the most important factors in the development of CAR-T treatment for solid tumors, the manufacture of bispecific CARs, addition of transcription regulator sequences, and combinational use with oncolytic viruses or chemicals, such as tyrosine kinase inhibitors, have been attempted since 2015 (Ghartey-Kwansah et al., 2018; Lynn et al., 2019; Mestermann et al., 2019; Miao et al., 2021). Therefore, we studied multifunctional synthetic CAR-T cells with improved cancer cell-killing efficacy for the effective treatment of solid tumors of the digestive system.

CLDN18.2 is transiently expressed in gastric epithelial cells and is rarely expressed in other normal tissues. However, CLDN18.2 expression is abnormally elevated in several cancerous tissues (Niimi et al., 2001). CLDN18.2 is expressed in gastric, esophageal, pancreatic, lung, ovarian, and bile duct cancers and in various other tumors. Accordingly, CLDN18.2 is considered a very promising target for treating CLDN18.2-positive tumors such as gastric and pancreatic cancers and is also recognized as a very challenging target for developing safe and effective transformed CAR-T cells (Morin, 2005). CLDN18.2-specific CAR-T cells are a promising treatment strategy for CLDN18.2-positive tumors. However, further efforts are required to increase the efficacy of solid tumor treatment and reduce the side effects of CLDN18.2-specific CAR-T cell therapy (Jiang et al., 2019; Shi et al., 2023).

Existing CAR-T cells have a limitation in that their activation in vivo cannot be controlled. CAR-T cells commonly cause fatal cytokine release syndrome (Rafiq et al., 2020; Xu et al., 2018). Additionally, the interaction between programmed cell death protein 1 (PD-1) expressed on activated T cells and programmed cell death ligand 1 (PD-L1) expressed on tumor cells may lead to the failure of T-cell therapy (Abate-Daga et al., 2014; Wei et al., 2019). In other words, T-cell activity is inhibited by the binding of the immune checkpoint proteins PD-1 and PD-L1, making it difficult to predict the efficacy of cancer treatments (Lei et al., 2020; Liu et al., 2016; Wei et al., 2019). Therefore, to overcome the problems associated with existing CAR-T cells, synthetic switch molecules must be developed to change the signal between T and target cells (Lorenzini et al., 2023; Yu et al., 2019). In addition to the development of these switch molecules, the development of CAR-T cell therapy with improved efficacy and safety that expands the treatment indications for solid cancers in addition to blood cancer is required (Liang et al., 2021; Weng et al., 2018; Xu et al., 2021). Moreover, researchers have developed one to four generations of CAR structures, of which the second-generation CAR has the structure of CD3ζ (T cell activation signal I) and costimulatory molecule CD28 or 4–1BB (T-cell activation signal II), which is most commonly used in the recent studies (Poorebrahim et al., 2019; Zuccolotto et al., 2021). We used inducible T-cell costimulatory (ICOS) (CD278) instead of the existing CD28 or 4–1BB for applying to solid tumors. In addition, by attaching synthetic PD1/CD28 to the CAR structure using an internal ribosome entry site (Li & Wang, 2020; Mazinani & Rahbarizadeh, 2022), we developed a CAR-T-cell therapy that overcomes the inhibition of T-cell activity by immune checkpoint proteins.

In this study, we designed three types of anti-CLDN18.2 CAR-T cells expressing an antigen-binding domain that specifically binds to CLDN18.2 and a switch molecule containing the extracellular domain of the PD-1 receptor and the intracellular domain of the CD28 receptor. CAR-T cells were produced using lentiviral vectors, and their functions and effects were analyzed.

## Materials and Methods

### Cell Lines and Culture Condition

Mammalian cells were maintained in medium supplemented with fetal bovine serum (Gibco), 100 units/ml penicillin, 100 μg/ml streptomycin, and 0.25 μg/ml amphotericin B at 37 °C and 5% CO_2_. HEK 293 (ATCC, CRL-1573) cells were grown in high-glucose Dulbecco’s modified Eagle’s medium (Gibco). NCI-N87, AGS, and KATO-III cells were purchased from GeneCopoeia. NCI-N87 cells were cultured in Roswell Park Memorial Institute 1640 Medium, AGS in Kaighn’s Modification of Ham’s F-12 Medium, and KATO-III in Iscove’s Modified Dulbecco’s medium. Claudin18.2 over-expressing cell lines, NCI-N87, AGS, and KATO-III lines were isolated through lentiviral vector transfection and selected under puromycin pressure.

### Designing of CAR-PD1/CD28 Constructs and Subcloning into Lentiviral Vector

Anti-Claudin18.2 CAR constructs were designed to include an antigen-binding domain, transmembrane domain, costimulatory domain, and chimeric-switch receptor (PD1/CD28). The DNA sequences for each domain and region in the anti-Claudin18.2 CAR constructs were chemically synthesized by BIONICS. The synthesized DNAs for CT-001 and CT-002 were digested with the restriction enzymes *XbaI* and *XhoI* and subcloned into the CARBioV1-MCS vector, which was modified from pLenti-EF1a-Backbone (NG) (Addgene). The EZ-FusionTM HT Cloning kit (Enzynomics) was used to subclone the CT-017 construct. The plasmid DNAs were assessed using electrophoresis, and the entire inserted DNA sequence was confirmed by sequencing analysis. Three plasmids carrying different combinations or sequences of anti-Claudin18.2 CAR construct elements were obtained and named pCT-001, pCT-017, and pCT-002.

### Lentivirus Production

Lentivirus carrying anti-CLDN18.2 CAR constructs were produced using the LV-MAX™ Lentiviral System (A35684, Thermo Fisher). Viral Production cells (Gibco) were transfected with the target CAR plasmid and three types of packaging plasmids according to the manufacturer’s instructions. At 48 h after the transfection, the culture medium was centrifuged at 1,300 × *g* for 15 min and then filtered through a 0.45-µm filter. The supernatant was transferred to a fresh tube to harvest the lentivirus. A Lenti-X concentrator (Takara, Cat# 631232) was used to concentrate the harvested lentiviruses. The supernatant with the concentrator was incubated at 4 °C for 24 h and centrifuged at 1,500 × *g* for 45 min. The produced lentivirus was aliquoted and stored at − 80 °C until use.

To investigate the titer of the produced lentivirus, HEK293 cells were transduced with the diluted lentivirus and incubated for 48 h. After transduction, the supernatant of the cell culture was harvested and the lentivirus titer was determined using flow cytometry.

### Generation of Genetically Modified CAR-T Cells

Human peripheral blood mononuclear cells were purchased from Zen-Bio. T cells were activated by incubating CD3 T cells with CTS Dynabeads CD3/CD28 (Gibco) at a 1:1 ratio. Two days after activation, Claudin18.2 CAR lentivirus was added at 3 multiplicity of infection to T cells in the presence of 8 μg/ml polybrene. At 24 h post-transduction, lentivirus was removed from the culture, and T cells were further cultured in CTS™ OpTmizer™ T Cell Expansion SFM (Gibco) supplemented with 5% human serum (Sigma) and 200 IU/ml interleukin 2 at 37 °C and 5% CO_2_. CAR expression levels were examined by staining with goat anti-mouse IgG F(ab’)2 (Jackson ImmunoResearch) using flow cytometry at 4, 6, 8, and 11 days after transduction.

### Flow Cytometry

A CytoFLEX flow cytometer (Beckman Coulter) was used. Claudin18.2 expression in cancer cell lines was evaluated by staining with Claudin18.2 recombinant human monoclonal antibody (Invitrogen) and goat anti-human IgG Fc secondary antibody-PE (Invitrogen). For the immunophenotyping of T cells and peripheral blood mononuclear cells, CD3-PerCP Cy5.5 (BioLegend), CD4-PE Cy7 (BioLegend), CD8-APC, CD14-FITC (BD), CD45-BV421 (BD), CD56-PE (BD), CD19-APC (BD), CD25-FITC (BioLegend), and CD69-APC Cy7 (BioLegend) antibodies were used. Data were analyzed using the Kaluza analysis software (Beckman Coulter).

### Cytotoxicity Assays

Human gastric cancer cells, NCI-N87, NCT-N87-C18.2, AGS, AGS-C18.2, KATO-III, and KATO-III-C18.2 (2 × 10^4^), were co-cultured with CAR-T cells and non-transduced T cells at effector to target (E:T) ratios of 0.3:1, 1:1, and 3:1 for 24 h. From the co-cultures, 50 μl of the supernatant was transferred into each well of a 96-well plate. Lactate dehydrogenase was detected in the supernatant of co-cultures using CytoTox 96® Non-Radioactive Cytotoxicity Assay kit (Promega). The cytotoxicity (%) was calculated using the following equation:

$$\mathrm{Cytotoxicity}\;\left(\%\right)=\left(e\mathrm{xperimental}-\mathrm{effector}\;\mathrm{spontaneous}-\mathrm{target}\;\mathrm{spontaneous}\right)/\left(\mathrm{target}\;\mathrm{maximum}-\mathrm{target}\;\mathrm{spontaneous}\right)\times100$$

### Cytokine Release Assay

NCI-N87, NCT-N87-C18.2, AGS, AGS-C18.2, KATO-III, and KATO-III-C18.2 (2 × 10^4^) cells were co-cultivated with CAR-T and non-transduced T cells at an effector: target (E:T) ratio of 1:3 for 24 h. The supernatant was collected from the co-cultivated cells through centrifugation. Cytokine interferon-gamma (IFN-γ) and tumor necrosis factor-alpha (TNF-α) were examined using the Human IFN-γ ELISA kit (R&D) and Human TNF-α ELISA kit (R&D) in the cell supernatant.

### In Vivo Experiments

Five-week-old female NOG mice were purchased from COATECH Co., Ltd. To establish NCI-N87 xenograft mice, 5 × 10^6^ cells of NCI-N87-CLDN18.2 were subcutaneously injected into NOG mice. The size of tumors at the cancer cell-injection sites and the body weight of the mice were measured twice per week (Kusakawa et al., 2015). Mice were divided into four groups (NTD-T, CT-001, CT-002, and CT-017 CAR-T) at 23 days post-injection. NTD-T and anti-CLDN18.2 CAR-T cells (CT-001, CT-002, and CT-017 CAR-T) were prepared at a concentration of 2 × 10^6^ cells/100 µl in cold phosphate-buffered saline. CAR-T and NTD-T cells were injected intratumorally (IT) into mice, and seven days after the first IT injection, CAR-T and NTD-T cells were injected intravenously into mice via the tail vein. The anticancer efficacy of CAR-T cells in vivo was evaluated by measuring the changes in tumor volume and body weight of mice. A bioluminescence imaging system was used for the in vivo assessment of the anticancer activity of CAR-T cells using an in vivo imaging system (IVIS) Spectrum Imaging System (Perkin Elmer). At 7, 12, 20, 23, 27, and 33 days post CAR-T cell administration, the mice were anesthetized using isoflurane. Luciferin was injected intraperitoneally 10 min before imaging at 200 µl/mouse as a luminescent substrate. Images were acquired from the lateral position of the mice using the luminescence mode. The total flux value (unit: photons/s) was obtained and assessed from a region of interest of the same size from whole-body images. Data were analyzed using Living Image Version 4.7.2.20319.

### Statistical Analysis

Data are presented as mean ± SD. Statistical significance was analyzed using post-hoc tests after one-way ANOVA. Statistical analyses were performed using GraphPad Prism 10 software. Statistical significance was defined as *, **, and *** for *p*-values < 0.05, < 0.01, and < 0.001, respectively.

## Results

### Construction and Generation of Synthetic CLDN18.2 CAR-T Cells

**Fig. 1:**
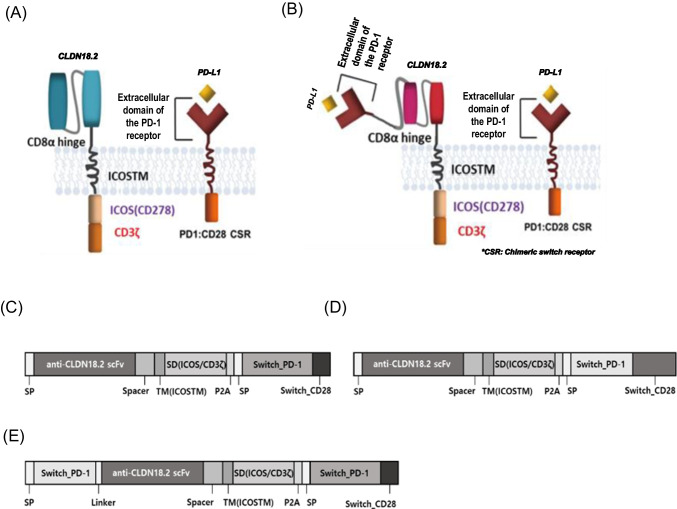
The structure of synthetic CLDN18.2 CARs. **A** and **B** Schematic illustrations of CAR structures of CT-001, CT-017 (**A**), and CT-002 (**B**). **C**–**E** Components of the synthetic CAR constructs CT-001, CT-017, and CT-002. Three individual CAR constructs include antigen-binding domain (anti-CLDN18.2 scFv), hinge domain (CD8α), transmembrane domain (ICOS), costimulatory domain (ICOS/CD3ζ), P2A, and chimeric-switch receptor (PD1/CD28) but have different composition and arrangement of transgenes and result in different sizes. The transgene sequences of the CT-001, CT-002, and CT-017 vectors are 2236, 2718, and 2352 bp, respectively. CT-001 and CT-017 have the same structure, except for the variation in the composition and arrangement of the PD1/CD28 CSR. CT-002 has an additional extracellular domain of the PD-1 receptor compared with CT-001. SP, signal peptide; TM, transmembrane domain; SD, stimulatory domain; P2A, 2A peptide; CSR, chimeric-switch receptor

**Fig. 2:**
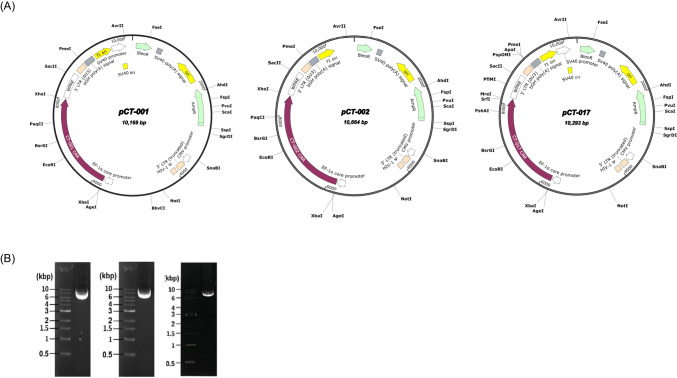
The subcloning of plasmids, pCT-001, pCT-002, and pCT-017. The DNA sequences of the self-designed CAR transgene were synthesized and subcloned into a third-generation lentiviral vector modified from the pLenti-EF1a-Backbone (NG). **A** Lentiviral plasmid maps of pCT-001, pCT-002, and pCT-017. For pCT-001 and pCT-002, the synthesized DNA fragments were subcloned into a lentiviral vector using *XbaI* and *XhoI*, while the EZ-FusionTM HT Cloning method was used to generate pCT-017. The total sizes of pCT-001, pCT-002, and pCT-017 are 10169, 10664, and 10293 bp, respectively. **B** Electrophoresis of plasmids, pCT-001, pCT-002, and pCT-017. Plasmids were prepared from each clone and examined using DNA electrophoresis. The insertions and sequences of the synthesized DNA fragments were confirmed by sequencing

We designed and synthesized three individual anti-CLDN18.2 CAR constructs, each having an extracellular antigen-binding domain (anti-CLDN18.2), hinge domain (CD8α), transmembrane domain (ICOSTM), costimulatory domain (ICOS/CD3ζ), P2A, and chimeric-switch receptor (PD1/CD28) (Fig. 1A, B). The CT-001, CT-017, and CT-002 constructs had different transgene compositions and arrangements, resulting in different sizes. CT-001 differs from CT-017 in the composition of PD1/CD28, and CT-002 has an additional extracellular domain of the PD-1 receptor compared with CT-001 (Fig. 1C, D, E). The transgene sequences of the CT-001, CT-002, and CT-017 constructs were 2236, 2718, and 2352 bp, respectively. To generate plasmids carrying the synthetic anti-CLDN18.2 CAR constructs, the designed CAR transgene DNAs were synthesized. We obtained plasmids pCT-001, pCT-002, and pCT-017 with sizes of 10, 169, 10,664, and 10,293 bp, respectively, by subcloning the synthesized CT-001, CT-002, and CT-017 construct DNA into a third-generation lentiviral vector modified from the pLenti-EF1a-Backbone(NG) (Fig. 2A). Plasmid DNAs were assessed using electrophoresis (Fig. 2B), and the insertion and sequences of each CAR construct were confirmed by DNA sequencing (data not shown).

**Fig. 3:**
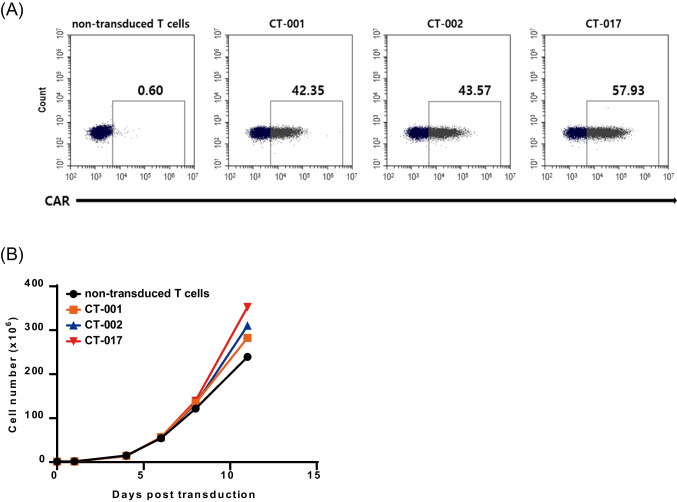
The characteristics of CLDN18.2 CAR-T cells. **A** Expression of CAR on T cells. Activated T cells were transduced using lentivirus encoding synthetic CLDN18.2-CAR constructs, and CAR expression level was assessed using flow cytometry after staining with FITC-labeled goat anti-mouse IgG F(ab’)2. **B** Proliferation curves of CAR-T cells and non-transduced T cells. In all cases, the cells expanded 200-fold 11 days post-transduction

We transduced T cells using lentivirus carrying synthetic anti-CLDN18.2 CAR constructs to produce genetically engineered CAR-T cells targeting CLDN18.2. The CAR expression was examined using flow cytometry using FITC-labeled goat anti-mouse IgG F(ab’)2. CT-001 CAR-T cells showed a CAR expression of 42.35%, CT-002 of 43.57%, and CT-017 of 57.93%. (Fig. 3A). We also assessed the proliferation rate of CAR-T cells. CT-001, CT-002, and CT-017 CAR-T cells and non-transduced T cells expanded approximately 200-fold after 11 days of lentivirus transduction (Fig. 3B).

### Cytotoxicity of CLDN18.2 CAR-T Cells against CLDN18.2-Positive Cancer Cell Lines In Vitro

**Fig. 4:**
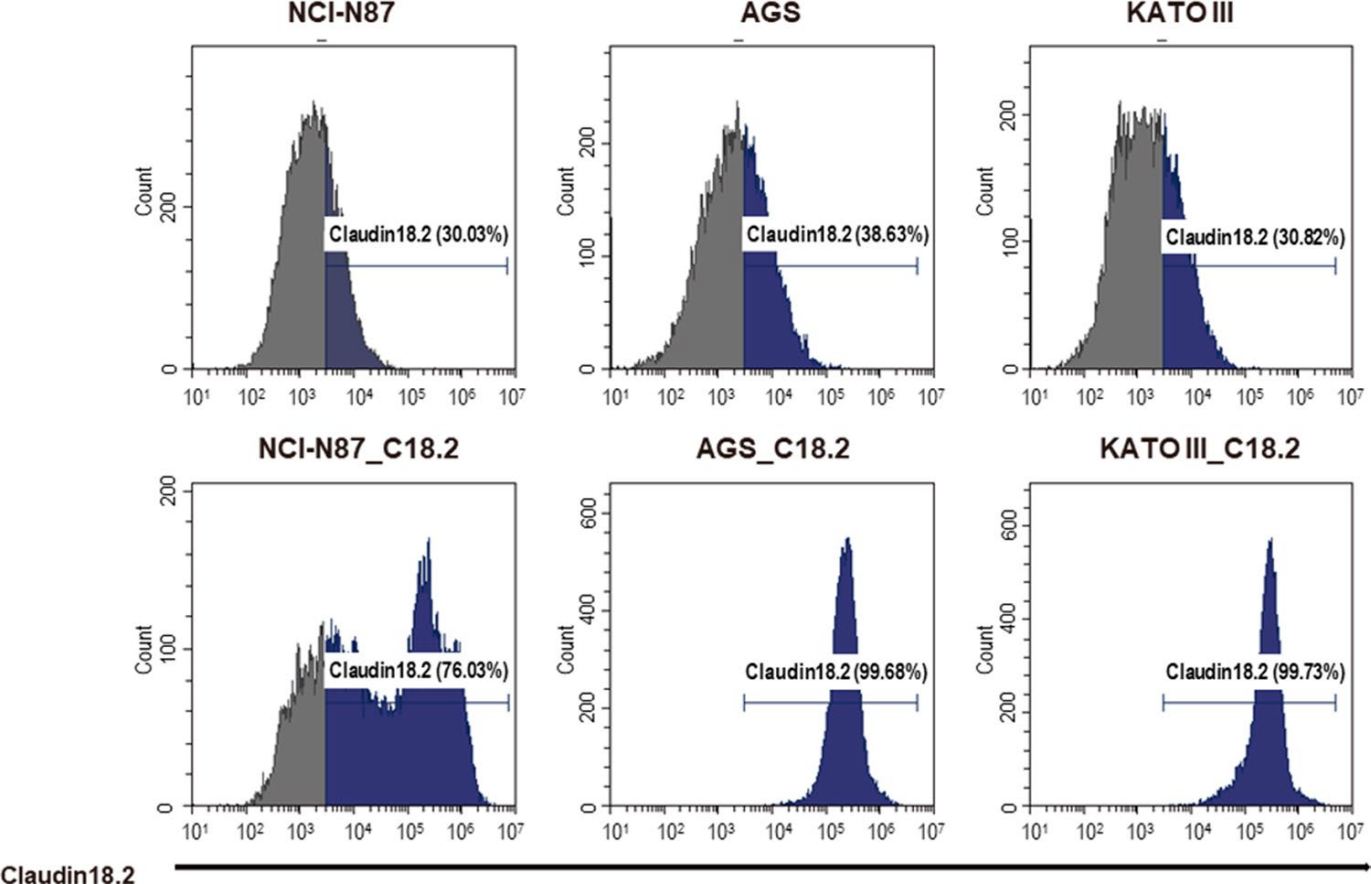
Expression of Claudin18.2 in cancer cell lines. Claudin18.2 expression on cancer cells was examined by staining with Claudin18.2 recombinant human monoclonal antibody (Invitrogen) and goat anti-human IgG Fc secondary antibody-PE (Invitrogen). Claudin18.2 expression levels were 30.03% in NCI-N87, 38.63% in AGS, and 30.82% in KATO-III. High expression levels of Claudin18.2 are shown in the Claudin18.2 over-expressing cell lines NCI-N87-C18.2 (76.03%), AGS-C18.2 (99.68%), and KATO-III-C18.2 (99.73%)

To address the specific activity of CLDN18.2 CAR-T cells against Claudin18.2 in vitro, we isolated Claudin18.2 over-expressing NCI-N87, AGS, and KATO-III cell lines. The expression of Claudin18.2 was assessed in NCI-N87, AGS, KATO-III, and Claudin18.2 over-expressing cell lines (Fig. 4). The overexpression of Claudin18.2 was confirmed in the NCI-N87-C18.2, AGS-C18.2, and KATO-III-C18.2 cells (Fig. 4). NCI-N87-C18.2 showed a CLDN18.2 expression of 76.03%, AGS-C18.22 of 99.68%, and KATO-III-C18.2 of 99.73%, while NCI-N87 showed CLDN18.2 expression of 30.03%, AGS, of 38.63%, and KATO-III, of 30.82% (Fig. 4).

**Fig. 5:**
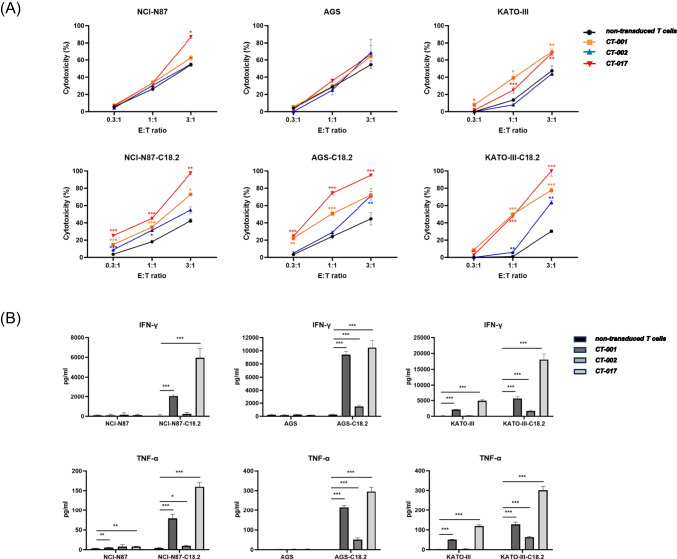
Cytotoxicity and cytokine release of CLDN18.2 CAR-T cells in the presence of Claudin18.2-positive tumor cells. **A** Target cells were co-cultured with different effector cells at the indicated E:T ratios for 24 h. Cytotoxicity was quantified based on the LDH release. Data represent the mean ± SD (*n* = 3), and *p*-values were calculated using post-hoc tests after ANOVA. **B** CLDN18.2 CAR-T cells were co-cultured with the target cells for 24 h, and the supernatants were collected for ELISA. Data represent the mean ± SD (*n* = 3), and p-values were calculated using post-hoc tests after ANOVA. *p < 0.05, **p < 0.01, ***p < 0.001

We assessed the cytotoxicity of CT-001, CT-002, and CT-017 CAR-T cells against gastric cancer cells. CLDN18.2 CAR-T, CT-001, CT-002, and CT-017 cells showed significantly higher cytotoxicity than non-transduced T cells in a dose-dependent manner in the presence of gastric cancer cells. CT-001 and CT-017 showed higher cytotoxicity at E:T ratios of 1:1 and 3:1 against all three CLDN18.2 positive gastric cancer cell lines. CT-001 CAR-T cells showed 35.19% cytotoxicity against NCI-N87-C18.2, 50.81% against AGS-C18.2, and 50.07% against KATO-III-C18.2 at an E:T ratio of 1:1, indicating twofold higher cytotoxicity for NCI-N87-C18.2 and AGS-C18.2, and approximately 50-fold higher cytotoxicity for KATO-III-C18.2 than non-transduced T cells. CT-002 CAR-T cells exhibited twofold higher cytotoxicity (31.49%) than non-transduced T cells against NCI-N87-C18.2, but no significant changes in cytotoxicity against AGS-C18.2 and KATO-III-C18.2, at an E:T ratio of 1:1. However, at an E:T ratio of 3:1, CT-002 also showed much higher cytotoxicity against all three CLDN18.2 over-expressing cell lines, resulting in twofold higher cytotoxicity against AGS-C18.2 (71.31%) and KATO-III-C18.2 (63.61%) (Fig. 5A).

A cytokine release assay was performed to determine the amount of cytokines released from CLDN18.2 CAR-T cells in response to gastric cancer cells. IFN-γ and TNF-α were investigated from the supernatant of co-cultured CAR-T and cancer cells. CT-001 and CT-017 released significantly more IFN-γ and TNF-α in the presence of NCI-N87-C18.2, AGS-C18.2, and KATO-III-C18.2 than non-transduced T cells. CT-002 CAR-T cells secreted more IFN-γ and TNF-α against AGS-C18.2 and KATO-III-C18.2 than non-transduced T cells. However, in the presence of NCI-N87-C18.2, CT-002 CAR-T cells induced increased secretion of TNF-α but not IFN-γ compared with non-transduced T cells (Fig. 5B). These results suggest that CLDN18.2, CT-001, CT-002, and CT-017 CAR-T cells have anticancer activity against gastric cancer cells in vitro. CT-001 and CT-017 showed greater activity than CT-002.

### In Vivo Experiments

**Fig. 6:**
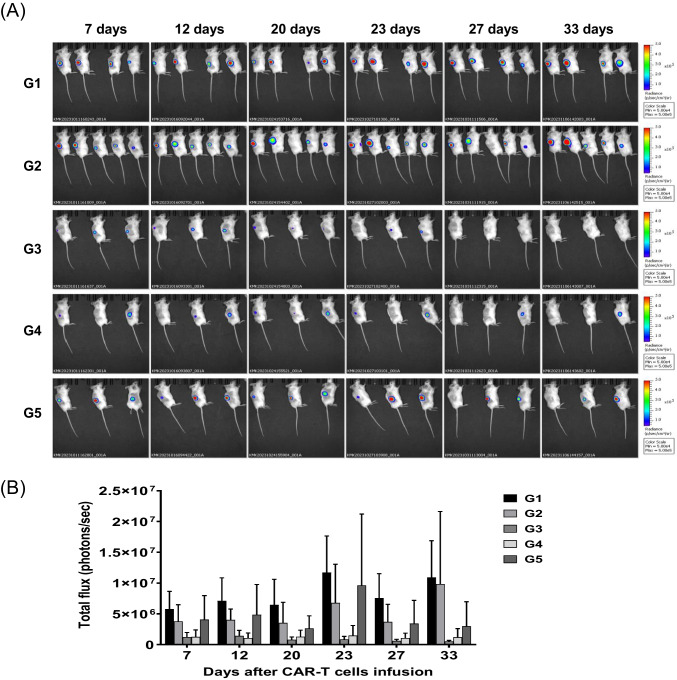
Bioluminescence imaging of mice injected with CAR-T cells. **A** The mice were imaged using an IVIS Spectrum in vivo imaging system (PerkinElmer). Bioluminescence was visualized in vivo after intraperitoneal injection of D-luciferin (15 mg/ml, 200 μl/head) and analyzed using the image software (Living Image Version 4.7.2.20319) of the imaging system. G1, non-transduced T-cell administration group; G2, untreated group; G3, CT-017 CAR-T cell administration group; G4, CT-001 CAR-T cell administration group; G5, CT-002 CAR-T cell administration group. CAR-T cells were injected intratumorally (IT) into xenograft mice. Seven days after the first IT injection, they were injected intravenously (IV) through the tail vein. Bioluminescence from cancer cells significantly decreased in the CAR-T cell administration group. **B** Quantification of the total flux of light in the luminescence images. The results obtained using the IVIS were analyzed after setting the region of interest (ROI) and obtaining the total flux ([photons/seconds]). Each bar represents mean ± SD (*n* = 3). G1, non-transduced T-cell administration group; G2, untreated group; G3, CT-017 CAR-T cell administration group; G4, CT-001 CAR-T cell administration group; and G5, CT-002 CAR-T cells. The reduction of cancer cells in the G3, G4, and G5 CAR-T cell treatment groups was confirmed

**Fig. 7:**
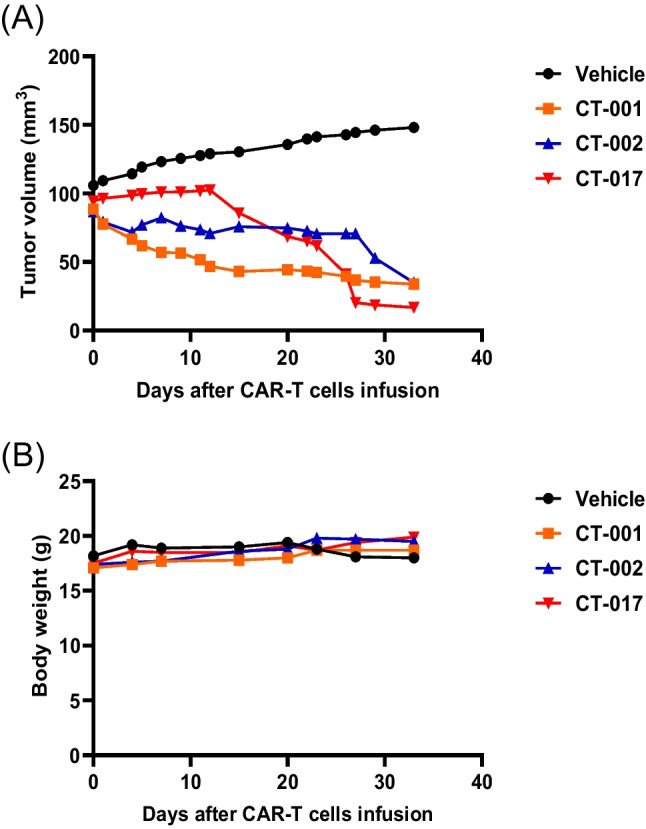
The changes in tumor volume in mice after infusion of CAR-T cells. **A** Tumor volume. **B** Body weight. Vehicle: untreated group; CT-001, CT-001 CAR-T cell administration group; CT-002, CT-002 CAR-T cell administration group; CT-017, CT-017 CAR-T cell administration group. It was confirmed that cancer cells in the CAR-T cell administration groups were significantly reduced, which was consistent with the results of an anticancer efficacy test using BLI performed at 12, 20 and 33 days after the administration of CAR-T cells. Considering that there were no changes, such as weight loss, in any of the groups, it was determined that the anti-CLDN18.2 CAR-T cells used in this experiment had no in vivo toxicity

The effectiveness of the anticancer effects following administration of anti-CLDN18.2 CAR-T cells was evaluated using an IVIS (Karimi et al., 2014) (Fig. 6). To assess the anticancer activity of CAR-T cells, 23 days after establishing the cancer-inducing animal model, 2 × 10^6^ CAR-T cells were injected IT, and after seven days, 4 × 10^6^ CAR-T cells were injected intravenously into the mice. The bioluminescence intensity of cancer cells in mice injected with CT-001 or CT-017 CAR-T cells decreased over time and significantly decreased 27 days after injection (Fig. 6). In addition, the tumor volume was measured at 3-day intervals from the time of tumor occurrence to the end of the experiment using a Digimatic caliper (Fig. 7). The results showed that the tumor volumes in each mouse were reduced in the CAR-T cell-administered group. Considering that there were no deaths or changes, such as weight loss, in the non-transduced T-cell administration group, the untreated group, and the anti-CLDN18.2 CAR-T cell administration group, it was determined that the anti-CLDN18.2 CAR-T cell therapy had no in vivo toxicity (Fig. 7). The anticancer efficacy of anti-CLDN18.2 CAR-T cell therapy was confirmed in vivo, and additional testing for clinical trials on patients will be possible.

## Discussion

We produced synthetic CLDN18.2-PD1/CD28 CAR-T cells to increase the antitumor efficacy of CLDN18.2 CAR-T cell therapy and verified their efficacy and safety in gastric cancer through in vitro and in vivo experiments. We designed CAR constructs containing ICOS (CD278) as a costimulatory factor and synthetic PD1/CD28 CSR to enhance anticancer activity of CAR-T cells. In addition, we expanded the indications for CAR-T cell therapy, which is mainly used to treat blood cancer, to solid cancers, designed and manufactured a new concept of CAR-T cell therapy capable of overcoming the inhibition of T-cell activity by immune checkpoint proteins, and confirmed its efficacy. Specifically, anti-CLDN18.2 CAR-T cells expressing a binding domain and a switch molecule that specifically binds to the cancer antigen CLDN18.2 were prepared.

In addition, CAR-T cells expressing a fusion protein of the PD-1 receptor, a CLDN18.2-specific antigen-binding domain, and a switch molecule were prepared. These are new concepts for manufacturing CAR-T cells based on the mechanism of action of PD-1 present on the surface of T cells and PD-L1 expressed on cancer cells. CAR-T cells have the advantage of overcoming the inhibition of T-cell activity by immune checkpoint proteins and have increased selectivity and reactivity to specific cancer antigens. The manufactured CAR-T cells exhibited excellent killing ability, specifically against cancer cell lines in which the CLDN18.2 gene was introduced. This result is consistent with a previous report showing that the PD-1 receptor is fixed to the cell membrane of CAR-T cells and prevents the inhibition of T-cell activity by binding to PD-L1 overexpressed on cancer cells (Li & Wang, 2020). We found that synthetic CLDN18.2-PD1/CD28 CAR-T cells activated the proliferation and differentiation of immune cells (e.g., T cells) due to the intracellular signaling action of the CD28 receptor and increased the secretion of cytokines such as IFN-γ and TNF-α which have immune response regulation and antitumor activities (Chen et al., 2021; Lorenzini et al., 2023). CAR-T cells are expected to effectively treat solid cancers, such as stomach, esophageal, pancreatic, and lung cancers.

In addition, synthetic CLDN18.2-PD1/CD28 CAR-T cells (CT-001) expressing an antigen-binding domain that specifically binds to CLDN18.2 and a switch molecule containing the extracellular domain of the PD-1 receptor and the intracellular domain of the CD28 receptor, have excellent cytotoxicity specifically against CLDN18.2 positive cancer cells. This result is consistent with the results that CAR-T cell therapy containing the synthetic PD-1/CD28 switch molecule can prevent the inhibition of T-cell activity due to the binding of PD-1 and PD-L1 and activate T-cell proliferation and differentiation by CD28; therefore, it has the advantage of increasing the complete remission rate for solid cancers, such as pancreatic and liver cancers, more effectively than conventional CAR-T cell therapy (Guo & Cui, 2020).

The corresponding mRNA CAR gene was synthesized using the same constitution as CT-001. The gene was cloned by inserting it into an in vitro transcription vector and an mRNA expression vector, and the cloned pDNA was linearized and then used as a template to perform mRNA in vitro transcription to produce mRNA CAR-T, which was introduced into T cells by electroporation. The cytotoxicity of mRNA CAR-T cells against cancer cell lines was verified using the Calcein AM assay (Invitrogen). In the case of Claudin18.2-CAR-T cells transduced with mRNA, 98.76% CAR-T cells were produced. In addition, CLDN18.2-CAR-T cells showed cytotoxicity against cancer cell lines expressing CLDN18.2, which was more than two-fold than that against cancer cell lines not expressing CLDN18.2. However, in animal experiments, the effect was relatively weak compared with that of CAR-T cells using viral vectors (data not shown). From these results, it was found that mRNA CAR-T cells are easy to manufacture but have stability problems, and further follow-up studies are needed to address these problems.

This study reports the results of research on synthetic CLDN 18.2 PD1/CD28 CAR-T cells for treating gastric cancer. Applying synthetic PD1/CD28 costimulatory receptors to CAR-T cells improves the efficacy of CAR-T cells in solid tumors. The efficacy and safety of synthetic CLDN 18.2 PD1/CD28 CAR-T cells in patients with advanced gastric cancer should be validated in future studies using patient-derived xenograft models and clinical trials.

In the future, the CAR-T cell technology can be used to develop cancer vaccines for recurrent pancreatic cancer and refractory malignant sarcoma along with disease-related neoantigen mRNA (Lin et al., 2022; Rojas et al., 2023). Currently, we are developing various genetically engineered CAR-T cells to enhance function using lentiviruses to treat incurable diseases, which is related to methods to improve the TME and overcome antigen heterogeneity (Ghartey-Kwansah et al., 2018; Lynn et al., 2019; Rafiq et al., 2020; Xu et al., 2018). In addition, the synthetic CAR-T cell technology we developed will establish the basic technology for the development of multifunctional CAR-T cells for the treatment of refractory and recurrent diseases and will further accelerate the development of new treatments.

## Data Availability

The datasets generated and analyzed during the current study are available from the corresponding author on reasonable request.
